# The efficacy and mechanism of berberine in improving aging-related cognitive dysfunction: A study based on network pharmacology

**DOI:** 10.3389/fnins.2023.1093180

**Published:** 2023-01-20

**Authors:** Jiuxiu Yao, Wei Wei, Jiayu Wen, Yu Cao, Hao Li

**Affiliations:** ^1^College of First Clinical Medicine, Shandong University of Traditional Chinese Medicine, Jinan, China; ^2^Wangjing Hospital, China Academy of Chinese Medical Sciences, Beijing, China; ^3^Department of Geriatrics, Xiyuan Hospital, China Academy of Chinese Medical Sciences, Beijing, China; ^4^Graduate College, Beijing University of Chinese Medicine, Beijing, China

**Keywords:** berberine, cognitive dysfunction, D-galactose, RT-qPCR, network pharmacology

## Abstract

**Objective:**

To analyze the effects and mechanisms of berberine in the treatment of aging-related cognitive dysfunction based on network pharmacology methods, molecular docking techniques, and animal experiments.

**Methods:**

A mouse model of cognitive dysfunction was constructed by subcutaneous injection of D-galactose (D-gal) for 10 weeks, and the neuroprotective effects of berberine on aging-related cognitive dysfunction mice were evaluated by the Morris water maze (MWM) and immunofluorescence staining. The targets of berberine were obtained by SwissTargetPrediction, GeneCards, and PharmMapper. Putative targets of cognitive dysfunction were obtained by GeneCards, TTD, and DrugBank database. The STRING database and Cytoscape software were applied for protein-protein interaction (PPI) analysis and further screening of core targets. The DAVID database was used for Kyoto Encyclopedia of Genes and Genomes (KEGG) and gene ontology (GO) enrichment analysis to clarify the biological processes and pathways involved in the intersection targets, and AutoDockTools was adopted for molecular docking verification of core targets. Finally, the core genes were validated using real-time quantitative PCR.

**Results:**

The MWM results showed that treatment with berberine significantly improved spatial learning and memory in mice with cognitive decline induced by D-gal. Immunofluorescence staining indicated that berberine modified the levels of aging-related markers in the brain. A total of 386 berberine putative targets associated with cognitive dysfunction were identified based on the public database. The core targets of berberine for improving cognitive function, include *Mapk1*, *Src*, *Ctnnb1*, *Akt1*, *Pik3ca*, *Tp53*, *Jun*, and *Hsp90aa1*. GO enrichment and KEGG pathway enrichment analyses indicated that the mechanism of berberine in the treatment of aging-related cognitive dysfunction is attributed to pathways such as PI3K-AKT and MAPK pathways. *In vivo* experiments further confirmed that *Akt1*, *Ctnnb1*, *Tp53*, and *Jun* were involved in the neuroprotective actions of berberine.

**Conclusion:**

This study reveals the multi-target and multi-pathway effects of berberine on regulating aging-related cognitive dysfunction, which provides preclinical evidence and may promote new drug development in mitigating cognitive dysfunction.

## 1. Introduction

Cognitive dysfunction, also known as cognitive impairment (CI), is characterized by memory loss, learning disabilities, and a decreased ability to concentrate on a particular task. The spectrum of CI can range from mild cognitive deficits that are not clinically detectable to dementia (Luchsinger, 2012). Dementia is usually diagnosed when acquired cognitive impairment becomes severe enough to impair social and/or occupational functioning (Hugo and Ganguli, 2014). The CI leads to a reduced quality of life in older adults and increases the risk of dementia and mortality (Park et al., 2013; Hu et al., 2020). The population aged 80 years and older is the fastest-growing segment of the global population, and prevention of age-related cognitive dysfunction is one of the greatest challenges facing healthcare today.

Mild cognitive impairment (MCI) is considered to be a state between normal cognitive aging and early dementia (Petersen, 2004). Notably, approximately 16% of subjects diagnosed with MCI returned to normal or near-normal cognition after approximately 1 year (Koepsell and Monsell, 2012). In a meta-analysis evaluating the rate of progression from MCI to dementia in 41 cohort studies stratified by population studies and clinical trials, more than half of the participants did not progress to dementia within 10 years, and the annual conversion rate for dementia and Alzheimer’s disease (AD) was approximately 7% (McGirr et al., 2022). In addition to the modification of some modifiable risk factors, treatment with traditional Chinese medicine (TCM) may play a role in the recovery of cognitive function.

Berberine (BBR) is an isoquinoline alkaloid, mainly found in the rhizome of *Coptis* sp., and the cortex of *Berberis* sp., and *Phellodendron* sp. Numerous studies have demonstrated multiple pharmacological effects of BBR, including anti-inflammatory (Jeong et al., 2009), anti-proliferative (Choi et al., 2008), hypoglycemic (Zhang et al., 2010; Pirillo and Catapano, 2015), hypocholesterolemic (Pirillo and Catapano, 2015), and anti-hypertensive effects (Bova et al., 1992). Previous studies have shown that BBR has antioxidant properties as well as protective effects against neurodegenerative diseases (Cheng et al., 2022), improving the cognitive decline associated with diabetes in db/db mice (Li et al., 2018). Recent studies have highlighted the anti-aging effects of BBR (Xu et al., 2017; Dang et al., 2020), which is considered to be one of the greatest risk factors for neurodegenerative diseases. Taken together, it will be of interest to explore the protective effects of BBR in neurodegenerative diseases.

**FIGURE 1:**
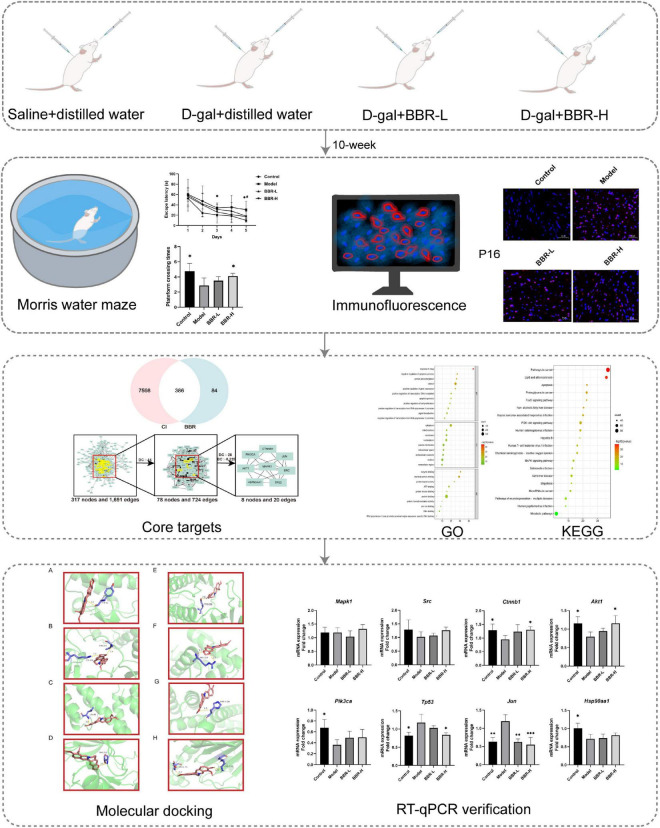
The flowchart of this study. Morris water maze: *p < 0.05 Control vs. Model; #*p* < 0.05 BBR-L/H vs. Model; RT-qPCR verification: *p < 0.05, ^**^p < 0.01, and ^***^p < 0.001 vs. Model.

D-galactose (D-gal) is an aldohexose naturally existing in the body, including in the brain (Nagy and Pohl, 2015). D-gal predisposes to aging and long-term systemic administration has been used to artificially produce brain aging phenotypes in animal models, which causes the onset of behavioral and cognitive deficits (Shwe et al., 2018). Several studies have illustrated that D-gal-induced brain aging not only contributes to memory deficits, neuronal degeneration, and apoptosis, but also increased oxidative stress, and mitochondrial dysfunction, which has many similarities to human brain aging (Banji et al., 2014). All these deficits eventually lead to cognitive decline. The amelioration of cognitive dysfunction by BBR has been observed in animal models of AD (Durairajan et al., 2012; Panahi et al., 2013). BBR has previously been observed to reduce the levels of endogenous oxidants and DNA damage response (Zhao et al., 2013), with potential effects of anti-aging (Dang et al., 2020; Li et al., 2022). However, the effects of BBR on cognitive impairment during aging have not been sufficiently investigated. In the current study, we investigated whether BBR reverses D-gal-induced aging and improves cognitive function in mice. The flow chart of this study is shown in Figure 1.

## 2. Materials and methods

### 2.1. Animals and general procedures

Eight-week-old male ICR mice (*n* = 32) were housed in eight cages. All animals were allowed free access to food and water and maintained at constant temperature (22 ± 3°C) and humidity (50 ± 10%) during a 12-h light/dark cycle. The project was authorized by the Ethics Committee of Xiyuan Hospital, China Academy of Chinese Medical Sciences (No. 2021XLC035-3).

After 1 week of adaptive rearing, D-gal-induced cognitive dysfunction was performed by subcutaneous injection of D-gal for 10 weeks according to a previously described procedure (Sun et al., 2018; Daroi et al., 2022). Similarly, BBR was administered orally as previously described (Ma et al., 2021; Wang et al., 2022) to observe the protective benefits against aging-related cognitive impairment. Mice were randomly divided into the following four groups (*n* = 8 per group). (1) Control: subcutaneous injections of saline and oral distilled water, (2) Model: subcutaneous injections of D-gal (150 mg/kg/d) and oral distilled water, (3) BBR-L: subcutaneous injections of D-gal (150 mg/kg/d) and BBR orally (50 mg/kg), (4) BBR-H: subcutaneous injections of D-gal (150 mg/kg/d) and BBR orally (100 mg/kg).

### 2.2. Drug

Berberine (BBR), purity ≧98% (LDSW220209-1), provided by Shaanxi Lande Biotechnology Co., Ltd. D- (+) galactose (ST1218-50 g) purchased from Shanghai Beyotime Biotechnology Co., Ltd. Pentobarbital sodium salt (P3761) was purchased from Sigma, USA.

### 2.3. Morris water maze test

The Morris water maze (MWM) experiment was conducted for 6 days during the 10th week of dosing. The water maze apparatus consisted of a circular pool (120 cm in diameter and 50 cm in height), an automatic camera, and a computerized analysis system. The pool was divided equally into four quadrants, and a 12-cm diameter platform was placed in the first quadrant and filled with an appropriate amount of water so that the top of the platform was 1 cm below the water’s surface. An appropriate amount of ink was poured into the water and mixed, and the water temperature was maintained at (23 ± 1)°C during the experiment. The MWM experiment consisted of two parts: navigation experiments and spatial probe experiments. On the day before the start of the experiment, each mouse was placed in the pool (without a platform) and swam freely for 90 s to adapt to the environment. The experiment started with 5 days of positioning navigation: each mouse was trained four times per day at 20-min intervals, with clockwise changes of entry points during the four training sessions. The mice were placed in the water facing the wall of the pool and the time between entering the water and finding the escape platform was recorded using a video tracking system, i.e., escape latency. If the platform could not be found for 90 s, the mice were manually guided to the platform for 10 s. On day 6, the spatial exploration experiment was started: the escape platform for the positioning navigation experiment was removed, and the mice were placed in the water facing the wall of the pool (the entry point was the midpoint of the third quadrant), and the number of times they crossed the original platform area within 90 s was recorded, i.e., the number of times they crossed the platform.

### 2.4. Tissue preparation

Brain tissue was taken after MWM. Mice were deeply anesthetized with an intraperitoneal injection of sodium pentobarbital, and the brain was quickly removed from the skull. Three animals in each group were randomly selected. Each brain was divided into left and right halves through a mid-sagittal incision, and the left one was fixed with 4% paraformaldehyde for performing immunofluorescence staining. The remaining cortex was snap-freezing in liquid nitrogen and then transferred to −80°C for further analyses.

### 2.5. Immunofluorescence staining

Paraffin sections were dewaxed in xylene and rehydrated in ethanol and distilled water. After antigen retrieval with EDTA, sections were blocked in 3% BSA for 30 min at room temperature. The blocked solution was gently shaken off and the primary antibody, namely, Anti -CDKN2A/p16INK4a Rabbit pAb) (GB111143, Servicebio, Wuhan, China) was added to the sections and incubated overnight at 4°C. The next day, a secondary antibody (Cy3 conjugated Goat Anti-Rabbit IgG, GB21303, Servicebio, Wuhan, China) was added and incubated at room temperature for 1 h. After DAPI (G1012, Servicebio, Wuhan, China) stained the nuclei, the sections were dried slightly and then sealed with anti-fluorescence quenched sealing tablets. Images of the cortex were captured at ×20 magnification with a fluorescence microscope (Nikon DS-U3, Nikon).

### 2.6. Key targets screening for BBR to improve cognitive dysfunction

#### 2.6.1. Identification of candidate targets for cognitive dysfunction

Three public disease-gene-related databases were used to retrieve important targets for Cognitive Dysfunction, including GeneCards^1^ (Safran et al., 2021), DrugBank^2^ (Wishart et al., 2018), and TTD (Zhou et al., 2022).^3^ The search term “cognitive dysfunction” or “cognitive impairment” is used to screen potential disease targets for cognitive dysfunction, and the disease targets obtained from the three databases were combined and de-duplicated. UniProt^4^ was used to standardize the gene names.

#### 2.6.2. Identification of candidate targets for BBR

To identify the corresponding targets of BBR, target prediction of BBR was performed through three public databases, including GeneCards (see text footnote 1) (Safran et al., 2021), SwissTargetPrediction^5^ (Daina et al., 2019), and PharmMapper^6^ (Wang et al., 2017), while all targets were restricted to Homo sapiens. The genes obtained from these three databases were aggregated and duplicates were removed. The UniProt (see text footnote 4) (UniProt Consortium, 2020) database was used to standardize the gene names.

#### 2.6.3. Construction of protein-protein interaction networks

Candidate target genes of BBR were intersected with CI-associated target genes to obtain intersecting genes and draw Venn diagrams, and these genes corresponding to both drugs and diseases were considered potential therapeutic targets. Online STRING 11.5^7^ (Szklarczyk et al., 2021) was used to construct protein-protein interaction (PPI) networks. All networks were built with Cytoscape v3.8.2, software for analyzing and visualizing biological networks. The core networks were analyzed based on degree, betweenness centrality, close-ness centrality, and average shortest path length. In the network, the nodes represent the targets, while the edges represent the connections between them.

#### 2.6.4. Enrichment analysis of GO and KEGG pathways

DAVID Bioinformatics Resource 2021^8^ (Sherman et al., 2022) was used to analyze gene ontology (GO) enrichment and Kyoto Encyclopedia of Genes and Genomes (KEGG) pathway enrichment for key targets involved in biological process, cellular component, molecular function, and signaling pathway. The bioinformatics online analysis platform^9^ was used to visualize the enrichment analysis results.

### 2.7. Molecular docking

The molecular structures of BBR were captured in the Traditional Chinese Medicine System Pharmacology database and Analysis Platform (TCMSP)^10^ (Ru et al., 2014) and the molecular structures (crystal structures) of the target proteins were obtained from the PDB database (Protein Data Bank).^11^ The molecular structures of BBR were captured in TCMSP (see text footnote 10) and the molecular structures (crystal structures) of the target proteins were obtained from the PDB database (Protein Data Bank, see text footnote 11). Pre-docking molecular processing was performed using AutoDockTools 1.5.7 software, and the pdbqt files were acquired. Molecular docking was performed using AutoDock Vina 1.1.2^12^ software, and the affinity score was calculated, with a smaller value indicating a stronger bonding force. PyMOL2.4.0 software^13^ was used to visualize the molecular docking results and calculate the Root Mean Square Deviation (RMSD) to verify the reliability, and the docking results were considered reliable with RMSD < 2A.

### 2.8. RT-qPCR

Total RNA was extracted from the mice’s cerebral cortex using an RNA extraction kit (Servicebio, Wuhan, China, G3640-50T). Reverse-transcription of RNA to cDNA was performed using ReverTra Ace qPCR RT Master Mix (TOYOBO, FSQ-201) according to the manufacturer’s instructions. Real-time qPCR was conducted on an ABI 7500 system using Applied Biosystems SYBR-Green PCR Master mix (Thermo Fisher Scientific, Inc., USA). The total reaction volume was 10 μL and primer sequences are shown in Table 1. The following amplification steps were used: initial denaturation at 95°C for 10 min, followed by 40 denaturation cycles at 95°C for 15 s, and elongation at 60°C for 60 s. *Gapdh* was used as a housekeeping gene. Relative mRNA expression was calculated using the 2^–ΔΔ*Ct*^ method. Three replicates were performed for each group.

**TABLE 1:** Primers used for quantitative real-time PCR.

Gene	Forward primer (5′-3′)	Reverse primer (5′-3′)
*Mapk1*	AACCTCCTGCTGAACACCACTT	CCACAGACCAAATATCAATGGACTT
*Src*	AGATCACTAGACGGGAATCAGAGC	GCACCTTTTGTGGTCTCACTCTC
*Ctnnb1*	GTTCTACGCCATCACGACACTG	AGGTCCTCATTATGTTTACTAAGGC
*Akt1*	TTTGGGAAGGTGATTCTGGTG	CAGGACACGGTTCTCAGTAAGC
*Pik3ca*	ATGGAGGAGAACCCTTATGTGAC	AGATTGAAAGGCAAAGGCGC
*Tp53*	CCCTCTGAGCCAGGAGACATT	CCCAGGTGGAAGCCATAGTTG
*Jun*	CCTTCTACGACGATGCCCTC	GGGTCGGTGTAGTGGTGATGT
*Hsp90aa1*	TTTACTCTGCCTATTTGGTTGCTG	CACAAAGAGAGTAATGGGATAGCC
*Gapdh*	CCTCGTCCCGTAGACAAAATG	TGAGGTCAATGAAGGGGTCGT

## 3. Statistics

Data were presented as mean ± SEM. MWM task data were analyzed using repeated-measures analysis of variance (ANOVA). The results of Mauchly’s test of sphericity were first used to determine whether there was a correlation between the repeated measures data and if there was a correlation (*p* ≤ 0.05), a multivariate ANOVA was performed, or the Greenhouse–Geisser correction was applied. Pairwise comparisons between different subgroups at the same time point were performed using multivariate ANOVA. For data that did not involve repeated measures, two independent samples *t*-test or Mann–Whitney *U*-test was used for analysis. *p* < 0.05 was considered statistically significant. All statistical analyses were performed with SPSS software version 26.0. GraphPad Prism 8 was used for generating data plots.

## 4. Results

### 4.1. Effects of BBR on cognitive dysfunction

**FIGURE 2:**
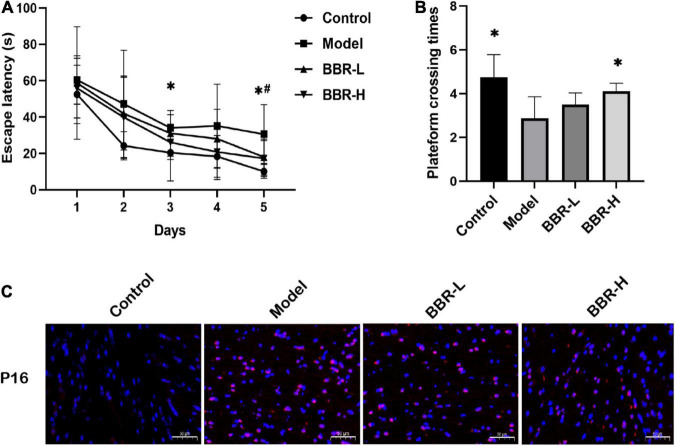
The results of Morris water maze (MWM) and immunofluorescence. **(A)** The escape latency. **(B)** The times across the platform (All data are mean ± SEM with *n* = 8, *p < 0.05 Control vs. Model; #*p* < 0.05 BBR-L/H vs. Model). **(C)** Immunofluorescence staining of P16 (×20, red = p16, blue = DAPI, scale bar 50 μm).

The MWM test is the most widely used laboratory behavioral test to assess cognitive deficits in rodents and the experimental protocol of MWM is as described previously (Vorhees and Williams, 2006). In the navigation test, as shown in Figure 2A, the escape latency of all mice gradually decreased with the increasing number of training sessions, indicating that their ability to locate the platform was enhanced. Compared with the control group, the escape latency of mice in the model group increased, with significant differences on days 3 and 5 (*p* < 0.05). The escape latency of mice in the BBR-L and BBR-H groups was diminished compared to the model group and exhibited a significant difference on day 5 (*p* < 0.05). In the exploratory experiment on day 6 (Figure 2B), the times of crossing the original platform were significantly reduced in the model group compared to the control group (*p* < 0.05). Both the BBR-L and BBR-H groups experienced an elevation in the number of mice crossing the original platform compared to the model group, with the BBR-H group showing a significant difference (*p* < 0.05). Collectively, these data suggest that we successfully induced aging-related cognitive dysfunction in mice, and more critically, we found that berberine ameliorated this cognitive impairment, although more studies are necessary to clarify the mechanisms involved.

### 4.2. BBR reduces the expression of P16 in brain tissue of cognitive dysfunctions mice

Aging is a common risk factor attributed to various neurodegenerative diseases, and aging of the brain eventually leads to CI. P16, a tumor suppressor gene can be considered the best biomarker of cellular senescence (Kim and Sharpless, 2006). Immunofluorescence staining of brain tissue for P16 at the end of administration to determine that D-gal caused aging-related cognitive dysfunction in mice. As shown in Figure 2C, P16 expression was significantly increased in the D-gal group compared with the control group, indicating that D-gal induced brain aging in mice. However, the administration of BBR altered the expression of senescence marker. Compared with the D-gal group, P16 expression was reduced in both the low- and high-dose groups of BBR. In conclusion, the above results suggest that BBR has anti-aging potential in the brain.

### 4.3. Results of network pharmacology study

#### 4.3.1. Target network analysis

To further elucidate the mechanisms and targets of BBR for improving cognitive function, we conducted a network pharmacology study. We applied three public disease gene-related databases, including GeneCards, DrugBank, and TTD, to retrieve major targets for CI/cognitive dysfunction. Ultimately, a total of 7,984 recognized CI-associated genes were explored (Supplementary Table 1). To identify the corresponding targets for BBR, we aggregated all predicted targets from GeneCards, SwissTargetPrediction, and PharmMapper. Finally, 470 targets for BBR were obtained (Supplementary Table 2).

#### 4.3.2. PPI network construction and core gene screening for BBR and CI

**FIGURE 3:**
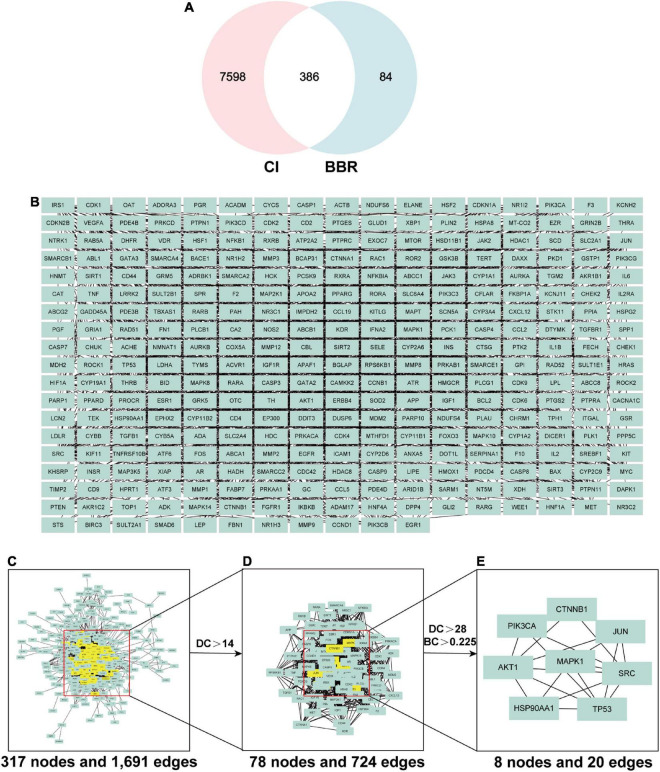
The screening process of core genes between cognitive impairment (CI) and berberine (BBR). **(A)** Venn diagram of CI and BBR overlapping genes. **(B)** Intersecting genes after screening. **(C)** Protein-protein interaction (PPI) network of intersecting genes. **(D)** PPI network of significant targets extracted from Panel **(C)**. **(E)** PPI network of candidate BBR targets for CI extracted from Panel **(D)**.

By intersecting candidate target genes of BBR with CI-related target genes, we identified genes intersecting with drugs and diseases as potential targets. The Venn diagram (Figure 3A) showed that there were 386 potential targets associated with both BBR and CI. Intersecting genes of BBR and CI were imported into the STRING database to construct the PPI network. We set the highest confidence level of 0.900 for the minimum required interaction score and hid the unlinked nodes in the network (Figure 3B). All network visualizations were implemented by Cytoscape. As shown in Figure 3C, the PPI network contains 317 nodes and 1,961 edges. Based on Degree and betweenness centrality, we selected the top 8 genes as hub genes (Figures 3C–E). These hub genes may represent the key molecular targets in the anti-CI action of BBR. Accordingly, eight core target genes were obtained (Figure 3E), including *Mapk1*, *Src*, *Ctnnb1*, *Akt1*, *Pik3ca*, *Tp53*, *Jun*, and *Hsp90aa1*.

#### 4.3.3. Enrichment analysis of BBR-CI target networks

**FIGURE 4:**
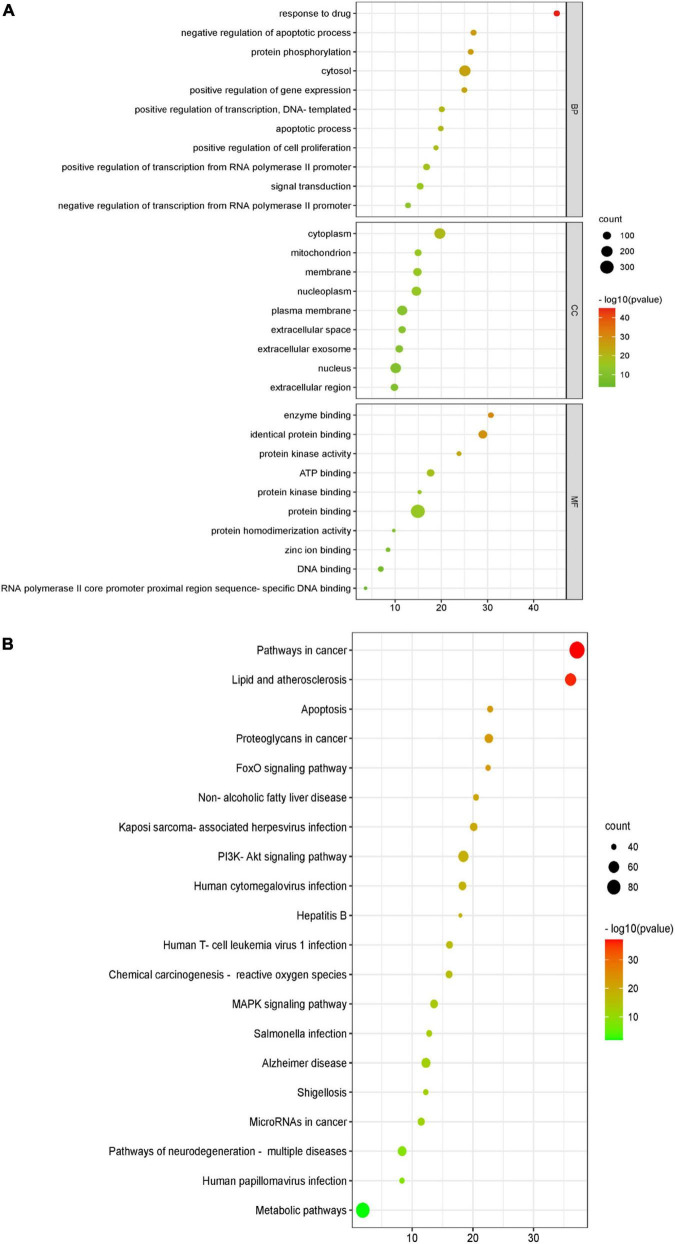
Gene ontology (GO) function and Kyoto Encyclopedia of Genes and Genomes (KEGG) pathway enrichment analyses of berberine (BBR) in the treatment of cognitive impairment (CI). **(A)** The GO function analysis, includes biological process (BP), cellular component (CC), and molecular function (MF). **(B)** Top 20 pathways in the BBR anti-CI bubble diagram of KEGG pathway enrichment.

Gene ontology (GO) analysis is commonly used to comprehensively characterize the contribution of genes in an organism, including biological processes, cellular components, and molecular functions. BBR therapeutic targets associated with CI were included in the analysis, and the results are shown in Figure 4A. KEGG is a database resource for understanding the high-level function and utility of biological systems from genomic and molecular level information. According to the KEGG pathway enrichment analysis (Figure 4B), the PI3K-Akt signaling pathway and MAPK signaling pathway were significantly enriched.

### 4.4. BBR-core targets molecular docking

**FIGURE 5:**
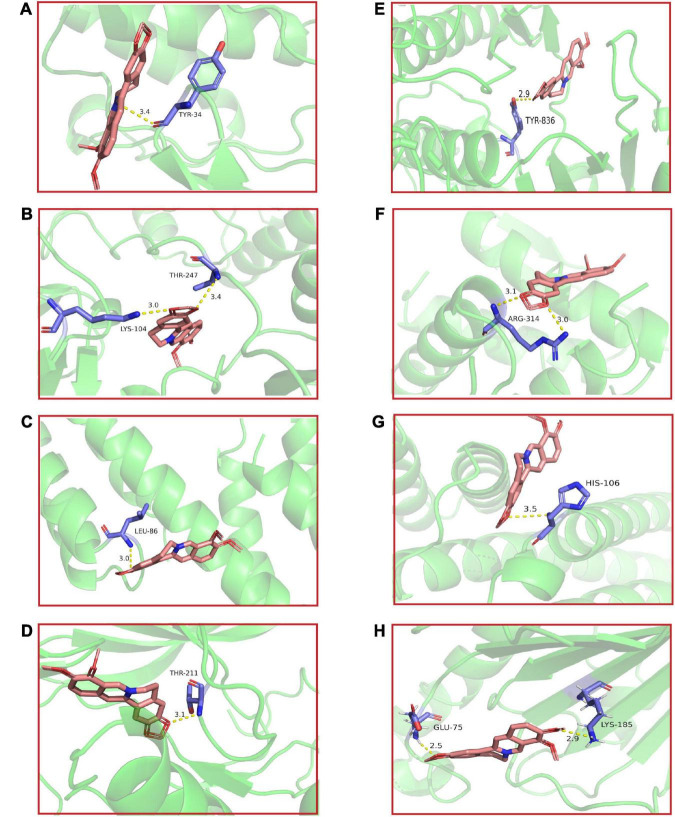
The molecular docking results of berberine (BBR) and key targets. BBR and **(A)** *Mapk1*; **(B)** *Src*; **(C)** *Ctnnb1*; **(D)** *Akt1*; **(E)** *Pik3ca*; **(F)** *Tp53*; **(G)** *Jun*; **(H)** *Hsp90aa1*.

Molecular docking of BBR and its corresponding core targets are achieved by AutoDock Vina software. It is generally believed that the smaller the affinity, the more stable the conformation of ligand-receptor binding and the higher the possibility of interaction. The molecular docking results demonstrated that BBR had a small affinity for all eight core targets (Table 2), indicating a favorable binding activity. The docking results are presented in Figure 5.

**TABLE 2:** Molecular docking results of 8 core targets and berberine (BBR).

Target name	Affinity (kcal/mol)
*Mapk1*	−7.6
*Src*	−9.6
*Ctnnb1*	−6.9
*Akt1*	−10.2
*Pik3ca*	−8.1
*Tp53*	−7.7
*Jun*	−6.9
*Hsp90aa1*	−7.0

### 4.5. RT-qPCR validation of core genes

**FIGURE 6:**
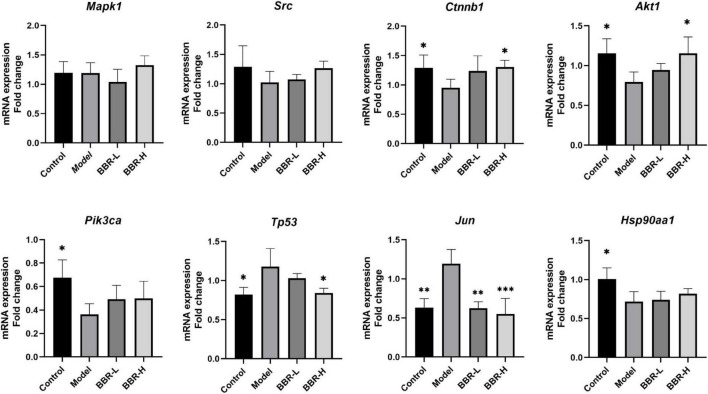
The results of RT-qPCR (All data are mean ± SEM with *n* = 3, *p < 0.05, ^**^p < 0.01, ^***^p < 0.001 vs. Model).

Further RT-qPCR was performed on brain tissues from mice in the four groups. The results (Figure 6) demonstrated that the expression of *Ctnnb1*, *Akt1*, *Pik3ca*, and *Hsp90aa1* was significantly lower in the model group compared with the control group (*p* < 0.05), the expression of *Src* showed a decreasing but not significant trend and the expression of *Tp53* and *Jun* was significantly higher (*p* < 0.05, *p* < 0.01, respectively). Compared with the model group, the expression of *Src*, *Ctnnb1*, *Akt1*, *Pik3ca*, and *Hsp90aa1* exhibited an increasing tendency in both the BBR-L and BBR-H groups, in which the expression of *Ctnnb1* and *Akt1* was significantly higher in the BBR-H group (*p* < 0.05). *Tp53* and *Jun* expression showed a decreasing trend and were significantly lower in the BBR-H group (*p* < 0.05, *p* < 0.001, respectively). Unfortunately, statistically significant results for *Mapk1* were not observed in our study.

## 5. Discussion

This study investigated the role and mechanism of BBR on aging-related spatial learning memory deficits due to D-gal with the assistance of network pharmacology and molecular docking techniques. Aging is considered to be the most important risk factor for neurodegenerative diseases, including AD. D-gal-induced aging mouse models have been widely used in aging studies, and long-term administration of D-gal induces aging, manifests neurological deficits, and shows significant spatial learning and memory deficits in the MWM test (Wei et al., 2008).

Based on a network pharmacological analysis, we constructed an animal model of aging-related cognitive impairment by subcutaneous injection of D-gal to evaluate the effects of BBR. Chronic administration of low doses of D-gal has been shown to induce changes that mimic the natural aging process in animals, including cognitive impairment (Wei et al., 2005). In our study, MWM behavioral tests were applied to detect changes in cognitive status after 10 weeks of subcutaneous injection of D-gal. We found that BBR treatment improved D-gal-induced learning and memory deficits. Previous studies have shown that BBR displays significant memory improvement activity in multiple animal models of memory deficits through various mechanisms, such as anti-inflammatory, anti-oxidative stress, cholinesterase inhibition, and anti-amyloid effects (Yuan et al., 2019). In addition, we explored the expression levels of aging markers and found that D-gal resulted in increased expression of the aging marker P16, and BBR administration ameliorated these changes. These results are consistent with previous studies (Dang et al., 2020). Administration of BBR for 6 months significantly improved cognitive deficits and insulin resistance in naturally aged rats (Yu et al., 2018). BBR emphasizes the induction of neuroprotective effects against Adriamycin-induced cognitive decline by modulating brain growth factors and exerting anti-inflammatory, anti-apoptotic, and antioxidant effects (Shaker et al., 2021). These results further support the ameliorative role of BBR in aging-related cognitive dysfunction.

Cognitive dysfunction is a common disorder in older individuals, and the incidence of MCI increases significantly with age (Petersen et al., 2018). MCI is a cognitive state between normal aging and dementia, and studies have shown that individuals with MCI are approximately three times more likely to progress to dementia over the next 2–5 years than age-matched controls (Petersen et al., 2018). It is well known that no drug is available to modify the disease process in AD, the most common type of dementia. Of particular note, it has been reported that 12.2% of MCI patients recover normal cognitive function within 3 years (McGirr et al., 2022). There are reasonable grounds to believe that reversing MCI to reduce the burden of dementia is a viable area of interest. There is compelling evidence to suggest that BBR was effective in improving cognitive function in Diabetes-related cognitive impairment and AD (Akbar et al., 2021; Hao et al., 2022). A spectrum of studies has demonstrated the ameliorative role of BBR in AD pathologies and cognitive dysfunction (He et al., 2017; Cai et al., 2018; Lin L. et al., 2020; Wu et al., 2021; Ye et al., 2021). BBR may improve cognitive impairment through a variety of mechanisms, including anti-inflammatory, anti-oxidative stress, improvement of insulin resistance, and inhibition of endoplasmic reticulum stress (Patil et al., 2015; Yu et al., 2018; Wang et al., 2019; Yuan et al., 2019; Shaker et al., 2021; Wu et al., 2021). Our study is the pioneer in exploring the mechanism by which BBR improves cognitive dysfunction with the assistance of network pharmacology and molecular docking techniques. To identify the therapeutic targets of BBR against CI, we identified 7,984 CI-validated targets and 470 BBR therapeutic targets using network pharmacology. We identified 386 potential targets associated with BBR and CI. In addition, BBR therapeutic targets associated with CI were used for GO and KEGG analyses. The results of GO analysis and KEGG analysis showed that BBR may exert its effects through MAPK and PI3K/Akt signaling pathways. Previous studies have shown that BBR can increase the levels of proteins located on neurosynapse through the MAPK signaling pathway (Zhou et al., 2016)and exert neuroprotective effects against tau protein hyperphosphorylation through PI3K/Akt pathway (Lin J. Y. et al., 2020).

The top 8 targets screened by network pharmacology were *Mapk1*, *Src*, *Ctnnb1*, *Akt1*, *Pik3ca*, *Tp53*, *Jun*, and *Hsp90aa1*. Molecular docking showed that the core targets dovetailed well with BBR, and RT-qPCR validated that the anti-aging-related cognitive dysfunction of BBR may be achieved by modulating *Akt1*, *Ctnnb1*, *Tp53*, and *Jun*. PI3K/Akt signaling pathway plays an essential role in regulating cell growth, proliferation, and survival. Akt activated by PI3K promotes cell growth and survival through the phosphorylation of multiple cytoplasmic proteins during senescence (Lin J. Y. et al., 2020). A previous study showed that berberine-induced PI3K/Akt activation exerts a protective effect by causing dephosphorylation of tau proteins and ameliorating neuronal axonal damage (Wang et al., 2018). In addition, BBR blocks Aβ production by activating the PI3K/Akt signaling pathway *in vitro* (Durairajan et al., 2012). Although our study only detected statistical differences in *Akt1*, *Pik3ca* showed the same trend of alteration, and it has also been shown that BBR can reduce tau hyperphosphorylation in 3 × Tg AD mice *via* the Akt/glycogen synthase kinase-3β (GSK3β) pathway (Chen et al., 2020). The *Ctnnb1* gene encodes β-catenin, a pivotal component of the Wnt signaling pathway that plays a significant role in the regulation of cell proliferation, differentiation, and apoptosis (Liu et al., 2022). Wnt signaling declines with age in the rat brain (Jessberger et al., 2009) and has been identified in the cerebral cortex of AD patients (Folke et al., 2019). Targeting recovery of the Wnt/β-catenin signaling pathway is a promising therapeutic strategy for AD (Jia et al., 2019). The *Tp53* gene produces the P53 protein, which functions as a transcription factor involved in cell cycle control, DNA repair, and regulation of cellular senescence and body aging (Rufini et al., 2013). A study reveals that BBR can depress the expression of P53 in primary neurons to keep cells in G0/G1 phase (Chai et al., 2013). *c-Jun* is an inducible transcription factor known to play a key role in neuronal cell death and survival (Raivich et al., 2004). Activated *c-Jun* expression is increased in the AD brain (Anderson et al., 1994; Raivich et al., 2004) and is present in the neurogenic fiber tangles of the AD brain (Pearson et al., 2006). Our results are in good agreement with previous studies and we support that BBR functions from multiple targets to improve cognitive function. In future studies, it is imperative to validate and explore our results more thoroughly and extensively.

## 6. Conclusion

Taken together, the results of this study suggest that BBR is able to attenuate spatial learning and memory impairment by inhibiting the expression of aging markers in the brain of the D-gal-induced senescence mice. Further network pharmacology and RT-qPCR validation suggest that this anti-aging effect may be achieved through the regulation of genes such as *Akt1*, *Ctnnb1*, *Tp53*, and *Jun*. These findings provide new evidence for the anti-aging protective effects of BBR and offer potential therapeutic directions for cognitive dysfunction. However, the specific targets of BBR in its anti-aging effects are not known, which is the main limitation of this study. Therefore, further studies are needed to identify whether BBR plays a role in improving cognitive function through improving the aging of specific cell types in the CNS and to elucidate the mechanism.

## Data availability statement

The original contributions presented in this study are included in this article/Supplementary material, further inquiries can be directed to the corresponding authors.

## Ethics statement

The animal study was reviewed and approved by Ethics Committee of Xiyuan Hospital, China Academy of Chinese Medical Sciences (No. 2021XLC035-3).

## Author contributions

JY and WW performed the experiments and data analysis and wrote the manuscript. JW assisted with the experiments. YC and HL assisted in conceptualizing and revising the manuscript. All authors reviewed and approved the manuscript.

## Footnotes

1
https://www.genecards.org/

2
https://go.drugbank.com/

3
https://db.idrblab.net/ttd/

4
https://www.uniprot.org/

5
http://swisstargetprediction.ch/

6
http://www.lilab-ecust.cn/pharmmapper/

7
https://string-db.org/

8
https://david.ncifcrf.gov/

9
https://www.bioinformatics.com.cn/

10
https://tcmsp-e.com/tcmsp.php

11
https://www.rcsb.org/

12
http://vina.scripps.edu/

13
https://pymol.org/2/
